# Melanoma Brain Metastases: A Systematic Review of Opportunities for Earlier Detection, Diagnosis, and Treatment

**DOI:** 10.3390/life13030828

**Published:** 2023-03-19

**Authors:** Michael Joseph Diaz, Isabella Mark, Daphnee Rodriguez, Beata Gelman, Jasmine Thuy Tran, Giona Kleinberg, Anna Levin, Alice Beneke, Kevin Thomas Root, Andrew Xuan Vinh Tran, Brandon Lucke-Wold

**Affiliations:** 1 College of Medicine, University of Florida, Gainesville, FL 32611, USA; 2 College of Medicine, University of Central Florida, Orlando, FL 32827, USA; 3 Department of Biology, University of Maryland, College Park, MD 20742, USA; 4 School of Medicine, University of Indiana, Indianapolis, IN 46202, USA; 5 College of Engineering, Northeastern University, Boston, MA 02115, USA; 6 School of Arts and Sciences, Rutgers University, Piscataway, NJ 08854, USA; 7 Department of Dermatology, Case Western Reserve University School of Medicine, Cleveland, OH 44106, USA; 8 Department of Neurosurgery, University of Florida, Gainesville, FL 32611, USA

**Keywords:** melanoma, cerebral metastases, MBM, early detection, systematic review

## Abstract

**Introduction:** Melanoma continues to represent the most serious skin cancer worldwide. However, few attempts have been made to connect the body of research on advanced melanoma. In the present review, we report on strides made in the diagnosis and treatment of intracranial metastatic melanoma. **Methods:** Relevant Cochrane reviews and randomized-controlled trials published by November 2022 were systematically retrieved from the Cochrane Library, EMBASE, and PubMed databases (N = 27). Search and screening methods adhered to the 2020 revision of the Preferred Reporting Items for Systematic reviews and Meta-Analyses guidelines. **Results:** Although the research surrounding the earlier detection of melanoma brain metastasis is scarce, several studies have highlighted specific markers associated with MBM. Such factors include elevated BRAFV600 mutant ctDNA, high LDH concentration, and high IGF-1R. The approach to treating MBM is moving away from surgery and toward nonsurgical management, namely, a combination of stereotactic radiosurgery (SRS) and immunotherapeutic agents. There is an abundance of emerging research seeking to identify and improve both novel and established treatment options and diagnostic approaches for MBM, however, more research is still needed to maximize the clinical efficacy, especially for new immunotherapeutics. **Conclusions:** Early detection is optimal for the efficacy of treatment and MBM prognosis. Current treatment utilizes chemotherapies and targeted therapies. Emerging approaches emphasize biomarkers and joint treatments. Further exploration toward preliminary identification, the timing of therapies, and methods to ameliorate adverse treatment effects are needed to advance MBM patient care.

## 1. Introduction

The incidence of melanoma has exponentially increased in developed countries, accounting for 1.7% of cancer diagnoses worldwide, and remains the fifth most diagnosed cancer in the United States [1]. Incidence rates vary across geographical locations. The annual incidence rates in regions with predominately fair-skinned populations have rapidly increased between 4 and 6% in North America, Northern Europe, and New Zealand, while countries in Asia have incidence rates that remain mostly unchanged [2]. These varying incidences are likely attributed to differences in pigmentation characteristics as melanoma is distinctly more prevalent among fair-skinned individuals and is rarely seen in darker-skinned individuals [2]. Sex and gender disparities also exist in melanoma as incidence rates are higher in men than women [3]. Although melanoma represents 1% of all skin cancers, it is responsible for over 80% of skin cancer deaths, signifying the importance of screening for those with risk factors involving family or previous history of skin cancer, congenital disorders, and UV exposure from predisposing lifestyle/profession [1]. Melanoma diagnoses are classified into five stages, 0–IV, where stage 0 is known as melanoma in situ, and the last stage is defined as metastatic melanoma (MM) [4]. If melanoma is detected in its early stages, there is a 99% 5-year survival rate for primary melanoma; however, this number significantly drops to 27% for metastatic melanoma (SD ± 5–10%) [5]. Visceral metastasis sites are associated with poor prognosis and survival in melanoma; the one-year survival rate is 36%, 13%, and 1% in patients with metastasis to one, two, or three different visceral sites, respectively [6].

Metastasis to the central nervous system (CNS) is a common occurrence for patients with advanced-stage melanoma, where up to 60% of all melanoma patients will develop brain metastasis during the progression of their disease [7]. The biological propensity for melanoma to migrate to the brain is not fully understood, but epidemiologic data implicate various factors that correlate to the development of melanoma brain metastasis (MBM); some factors include male sex, primary tumor site on the trunk, and histopathologic diagnosis of primary superficial spreading [6]. The diagnosis of MBM often results in dismal outcomes associated with the rapid decline in the quality and quantity of life, with the median overall survival (OS) from diagnosis of around 4–6 months [8]. Davies et al. reported a median survival rate of 5.92 months following 1995 (N = 123, *p* = 0.01, 95% CI [0.60, 0.94]) [9]. Previously, the treatment options for patients with MBM were limited to chemotherapy, whole-brain radiation therapy, stereotactic radiosurgery, and surgical resection [10]. However, the further development of targeted therapy, immunotherapy, and radiation therapy offers promising strategies and investigations to improve the outcome, treatment, and understanding of brain metastases [8,10]. The Food and Drug Administration approved checkpoint inhibitors and BRAF/MEK-targeted therapy in 2011 due to their enhanced survival benefits. As of 2016, the median survival of unresectable or metastatic melanoma patients was nearly two years with these therapies, while traditional chemotherapy ranged between 6 and 9 months [10]. Middleton et al. that found patients treated with temozolomide had a median survival time of 7.7 months and those administered temozolomide and dacarbazine had a lower median time at 6.4 months (95% CI [0.92, 1.52] [11]. Despite these advancements in the treatment for metastatic melanoma, MBM remains associated with high morbidity and mortality and poor median survival duration. The weighted median overall survival was recently reported between 5 and 9 months for single-agent chemotherapy, immunotherapy alone, or targeted therapy. These findings reflect the need for further studies on brain metastases and treatments to improve disease outcomes and extend the lifespan of patients with MBM [11,12]. 

This study aimed to review and summarize the detection and diagnosis of melanoma brain metastases, discuss the current treatment options, and highlight emerging clinical evidence and focus areas for future research. 

## 2. Methods

**Figure 1:**
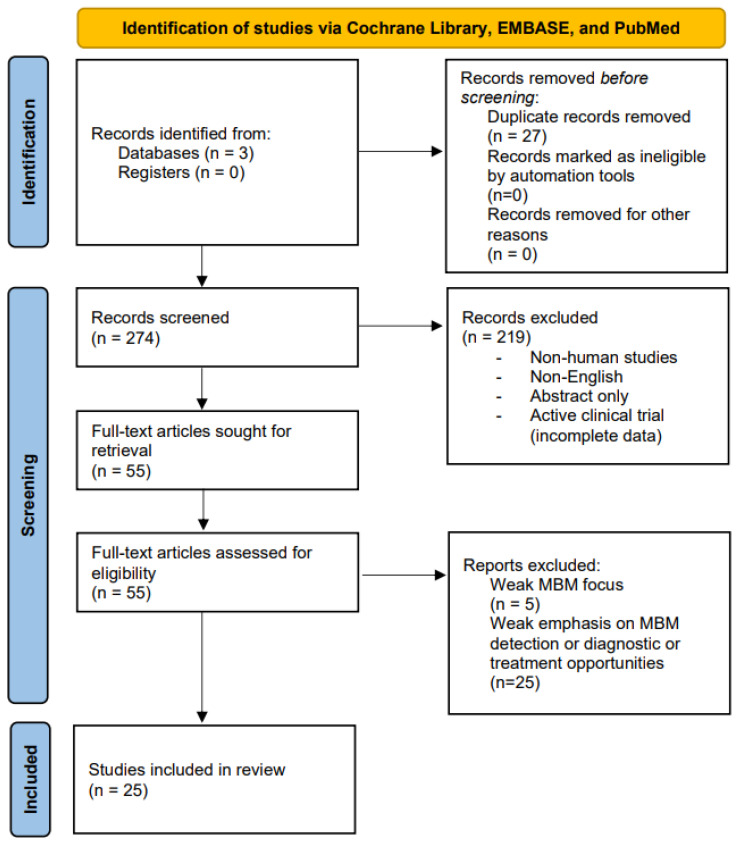
PRISMA flow diagram (2020 Revision) for the present systematic review.

A systematic query of the Cochrane Library, EMBASE, and PubMed was performed from database inception to 29 November 2022. The search and filtration processes were conducted in accordance with the current Preferred Reporting Items for Systematic reviews and Meta-Analyses (PRISMA) guidelines (Figure 1) [13]. Criteria for inclusion were (1) primary scientific focus on intracranial metastatic melanoma; (2) evidence-supported reporting of early detection, diagnosis, and/or treatment protocols for melanoma that has metastasized to the brain; and (3) reputable discussion of future directions for MBM research. Abstracts and articles that were not originally written in English were excluded. To limit methodological biases, only full-length articles archived in the Cochrane Database of Systematic Reviews and randomized-controlled trials were included. Ongoing clinical trials with yet-published results were excluded. Keyword search for peer-reviewed journal articles pertinent to our described focus (e.g., “intracranial melanoma” AND “detection”) returned a total of 301 eligible results (36 from Cochrane Library, 57 from PubMed, and 208 from EMBASE). After manual removal of duplicate results and filtration in accordance with our defined inclusion and exclusion criteria, we retained a total of 27 Cochrane reviews and randomized-controlled trials for review in the present study. Authors MJD, IM, and AB defined the inclusion and exclusion criteria. Authors DR, BG, JTT, and GK conducted the literature search and filtration processes. Authors MJD and KTR resolved any article selection disputes. 

## 3. Results

### 3.1. Detection and Diagnosis of Melanoma Brain Metastases

Metastatic melanoma that travels to the brain is a severe outcome of melanoma and is often given a poor prognosis [14]. Patients with metastatic brain melanomas have very high mortality rates, and the median survival rate after diagnosis is six months [15]. Additionally, in many cases, brain metastases are only detected in the later stages of cancer and are usually made up of multiple lesions [16]. The location of the metastasis often makes surgical resection difficult, which is compounded when the number of lesions is greater and when the size of the lesions is smaller. Moreover, even the effectiveness of treatment for a single or solitary brain metastasis is unclear and varies by patient [16]. Presymptomatic contrast CTs or MRIs are the primary method to identify CNS lesions. This identification is followed by characterization, conventionally achieved by the use of the Breslow thickness index and Clark level staging system as well as the notation of its subtype, mutation status, and anatomical location. The Breslow thickness index measures how vertically deep the tumor penetrates the skin layers to foretell the general likeliness of it spreading. The Clark scale is a 5-level scale that similarly specifies the number of skin layers a tumor invades. Though not fully comprehensive or infallible, these techniques are reliable for melanoma diagnoses/prognoses. As for determining the most effective treatment, however, there is still no reliable biomarker for MBM [15]. Studies have also not shown how these biomarkers may change throughout treatment and how they may signify improvement [16,17]. 

A promising biomarker is cell-free circulating tumor DNA (ctDNA) in determining the prognosis. A study revealed through quantitative PCR that the elevated pretreatment BRAFV600-mutant ctDNA concentration was associated with worse survival rates; conversely, low ctDNA and high lactate dehydrogenase (LDH) are associated with better survival rates when measured at week four, as demonstrated by univariate analysis (*p* < 0.0001) [17]. Evidence is increasingly demonstrating that pretreatment ctDNA, which is undetectable, is associated with significantly longer progression-free survival and overall survival [17]. Syeda et al. found that detectable ctDNA is associated with LDH concentration, the number of metastatic sites, the median sum of lesion diameters, and increased tumor burdens [17]. These results suggest that BRAFV600-mutant ctDNA could function as a predictive biomarker for overall and progression free survival prognosis. Furthermore, patients treated with dabrafenib plus trametinib, which maintained normal LDH levels and fewer than three organ sites with metastases, had the longest progression-free survival and overall survival [18]. Conversely, patients with more than two times the normal LDH concentrations had the shortest progression-free survival and overall survival rates [18]. Moreover, the patients with disease progression and new CNS lesions reported worse outcomes with a median survival time after progression of 4 months (n = 171) [18]. Due to the severe prognoses associated with metastatic brain melanoma, many studies often exclude this patient population from their studies. However, one study specifically examined melanoma patients with brain metastases and assessed the risk factors associated with the progression of their disease. Eastern Cooperative Oncology Group (ECOG) scores are frequently used by oncology health care professionals to assess the performance status of a patient in terms of functional status and ability for self-care for directing treatment decision and prognosis, where the scores are between 1 and 5, indicating increasing levels of disability [19]. Progression-free survival (PFS) is the duration of time between the initiation of a treatment to the progression of a disease or worsening, and this measure is used to determine how well a treatment works [20]. The results indicated that the following factors were associated with lower progression-free survival: ECOG ≳1, elevated serum LDH ≳three metastatic sites, and non-naive status [21]. Conversely, patients with ECOG 0, less than three metastatic sites, and LDH under the normal limits, typically had the highest progression-free survival (PFS) after six months [21]. Table 1 captures these factors and other predictors.

Additional risk factors have been assessed for the development of brain metastasis. Despite the previous proposal, a recent study found that an ulcerated primary was not associated with an increased risk of CNS progression [15]. When comparing patients with macroscopic lymph node involvement (or in-transit metastases) and patients with lymph node micrometastases, there was no significant difference in the CNS relapse rates across both groups [15]. Patients in stage IIIC were also more likely to have CNS progression than stage IIIA or IIIB [15]. In a comparable study, 10% of patients with stage III disease were at risk for, and up to 46% of stage IV patients will develop brain metastasis [15]. 

An additional study utilizing patient-derived xenografts (PDX) and live tissue samples established several biomarkers that may be implicated in MBM diagnosis. Notably, tumors from patients given therapy displayed significantly higher levels of IGF-1R than those from patients without therapy [22]. This finding supports previous findings that IGF-1R signaling may confer melanoma resistance to BRAF inhibitors, which suggests that the increased tyrosine kinase receptor expression modulates a response in cancer treatments [22]. Accordingly, samples with BRAF hotspot mutations were significantly more concurrent and deleterious than other mutations overall [22]. PDX derived from patients with stage IIIB/C melanoma showed spontaneous metastasis in animals to be independently correlated with patient outcomes [22]. A 2013 study tested the capacity of whole-body diffusion-weighted imaging (WB-DWI) alone to detect metastases in a separate cohort of 19 advanced melanoma patients compared to WB-DWI plus contrast-enhanced magnetic resonance imaging [23]. The results indicated that although WB-DWI alone was powerful for extracranial metastasis, several brain metastases were undetected [19]. Still, as Petralia and colleagues demonstrated, albeit a decade ago at the time of writing, we can do well with less [23].

### 3.2. Treatment of Melanoma Brain Metastases

**Figure 2:**
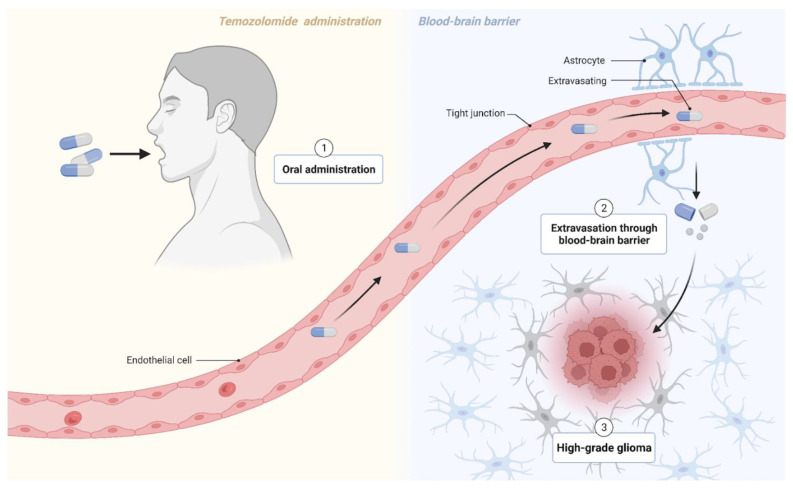
Temozolomide crosses the blood–brain barrier to target high-grade glioma cells. Figure created with BioRender.com (accessed on 17 December 2022).

Although current cancer treatment paradigms may reflect any combination of surgery, chemotherapy, radiotherapy, and immunotherapy with subsequent medical management, the options for treating melanoma brain metastases remain limited. As per usual practice, neurosurgery is a viable option for patients with solitary or single brain metastasis and for those desiring symptomatic relief. These surgical resections must still be followed by radiotherapy or a drug regimen that targets clinically undetectable micrometastases that likely remain elsewhere in the brain [24]. Regardless of surgical candidacy, systemic agents such as chemotherapeutics are prescribed to metastatic patients. However, such options fall short for MBM cases because penetration through the blood–brain barrier complicates drug delivery. Of the chemotherapy drugs that can enter the brain, they still only show modest efficacy [25]. As such, a recent push to unveil therapeutic targets that do not evade MBM treatment has spurred a burst of novel immunotherapy options. Several labs have looked at two new classes of drugs, immune checkpoint inhibitors and small-molecule targeted drugs, as alternatives to conventional chemotherapy. Both have significantly improved progression-free survival rates and patient prognosis of stage IV melanoma patients with brain metastases [25]. For example, previous research has cited that the co-administration of temozolomide (TMZ) and IFN-α2b exhibits antitumor activity, increased response rates, and in some instances, complete remission [26]. Temozolomide with sorafenib also showed antiangiogenic effects [27]. In vitro studies have also reported synergistic antitumor activity from TMZ with cisplatin. Even more promising, numerous phase III trials of a TMZ-based regimen administered to melanoma patients reported a reduction in CNS metastases from developing, proposing that the drug has prophylactic potential in patients with melanoma [28]. Therefore, temozolomide exposure is an attractive and encouraging treatment option that should be considered not only for first-line brain metastasis treatment, but also for early-stage melanoma presentation (see Figure 2).

Furthermore, evidence on the efficacy of ipilimumab and nivolumab, immune checkpoint inhibitors of PD-L1, is conflicting. Gritsch et al. reported a correlation between these agents and longer recurrence-free survival (RFS) and distant metastasis-free survival (DMFS) in MBM patients [29]. In the same year, Guo et al. claimed that PD-1 inhibitors had questionable long-term neurological and cognitive sequelae; the drugs could potentially exacerbate radiation-related necrosis [30]. High-dose injections of Allovectin-7, on the other hand, have been shown to be well-tolerated in stage III/IV MBM patients, but the results are lesion-specific [31]. Others have explored IFN-α and interleukin-2 (IL-2) both independently and jointly. Controversially, Li et al.’s randomized control trials brought them to the conclusion that subcutaneous INF-α injections are safer than IL-2 injections, but IL-2 is more effective at eradicating tumors and for relapse-free outcomes. Although IL-2 had greater brain tumor responses, it also induced more severe toxicities elsewhere in the body, ultimately diminishing the patients’ quality of life and posing a threat for their overall survivability [32]. Despite some ambiguities and the warranting of more testing, there is overwhelming evidence of immunotherapeutic (IT) approaches being key to formulating an effective management plan for complete tumor response in MBM. 

For optimal success rates, IT is combined with radiation. Traditionally, two forms of radiotherapy are deferred to—whole-brain radiotherapy (WBRT) and stereotactic (SRS) radiosurgery—for treating metastatic spread to the CNS. SRS has recently gained traction, whereas WBRT has also proven to be wholly unpromising for treating MBM. A comprehensive look into past research revealed the role of WBRT as controversial, failing to consistently prove benefits for treating MBM [18,33]. Moreover, some contend that WBRT is radioresistant and that unnecessary exposure to radiation toxicities worsens patient neurocognitive function. Specifically, patients receiving WBRT showed a neurocognitive decline in the form of memory loss and impaired executive function [24]. Manipulating WBRT dosage also showed no difference in effect on brain tumor size change or overall survivability, again indicating that WBRT is most likely not a favorable treatment approach [34]. Though most reporting on and the analysis of WBRT efficacy in MBM cases align with the aforementioned negative findings, a select few studies have seen WBRT improve palliation by prolonging both intracerebral and intracranial control [35,36]. Additionally, Janavicius and colleagues promoted WBRT as a means of preserving memory and neurocognitive function in MBM patients, which is in direct contention with earlier findings. However, Janavicious’s research group simultaneously ceded to WBRT critics, admitting that WBRT does not appear to benefit the survival outcomes [24,37]. For these reasons, this treatment modality is currently deemed as controversial. At best, there may be some narrow indications for WBRT, but by and large, WBRT seems to provide a much greater risk than reward. 

The second form of radiotherapy, stereotactic radiosurgery (SRS), has experienced a rapid uptake among patients with brain metastases. It has become the preferred radiotherapeutic option for numerous reasons. In SRS, external ionizing radiation beams are precisely focused on metastatic tumors, minimizing toxicity exposure. SRS has been demonstrated to be well-tolerated and to extend survivability when paired with immunotherapy [36,37,38,39]. Alone, SRS does provide a durable early response, but the effects are minimal when not administered concomitantly with IT [29].

Among the described treatment methods, the combined treatment of SRS and immunotherapy for MBM yields the best treatment pattern outcomes, and presently, it is the most scientifically supported therapeutic approach for targeting MBM. The most optimal results for OS and PFS are derived from conjunctive treatments via immunotherapies and radiotherapies in patients with MBM. Future work should pursue the optimal combination ratio and timing of the two therapies. 

### 3.3. Emerging Clinical Evidence and Focus Areas

There is a variety of emerging clinical evidence relevant to melanoma brain metastases. A large quantity of new research seeks to identify and improve novel and established treatment options. Emerging trials showcase how newly tested combinations of medications can increase the clinical efficacy for metastatic melanoma. For example, the characterization of nivolumab and ipilimumab therapy is ongoing as the effects still need to be fully understood, especially in active brain metastases. A phase II trial led by Long achieved significant intracranial response in patients with active melanoma brain metastases [40]. With no treatment-related deaths, intracranial response was achieved across three patient cohorts, with 46% (95% CI, 29–63) in the first cohort showing intracranial response, followed by 20% (95% CI, 7–41) in the second cohort, and 6% (95% CI, 0–30) in the third cohort [40]. Complete intracranial response was stimulated across the three patient cohorts at rates of 17%, 12%, and 0%; however, grade 3 or 4 treatment-related adverse events occurred in all three cohorts in 54%, 16%, and 13% of patients, respectively [40]. Despite treatment-related adverse effects, joint nivolumab and ipilimumab therapy showed a high intracranial response, indicating its potential as a primary therapy option for untreated and asymptomatic melanoma brain metastases [40].

Since Long et al.’s contributions, a smattering of other encouraging data points has surfaced. Di Giacomo et al. recently presented the first phase III trial exploring the improved survival of patients with asymptomatic brain metastases following ipilimumab treatment alongside fotemustine or nivolumab treatment [41]. Their results indicated that treatment with ipilimumab and nivolumab provided the highest four-year survival rate of the tested combinations at 41% (95% CI, 20.6–61.4) [41]. More recently, Larkin et al. confirmed that independent nivolumab treatment maintained superior efficacy compared to independent ipilimumab treatment for patients enrolled as per the American Joint Committee on Cancer version 8, similar to version 7 criteria [42]. A focus on the further characterization of dual treatment versus the independent treatment of both drugs for patients diagnosed by the latest criteria is still necessary to identify a treatment course with the highest clinical efficacy. In their own trial follow-up, Tjulandin et al. identified prolgolimab, an lgG1 anti-PD-1 monoclonal antibody, as another therapy option for non-resectable, metastatic melanoma patients [43]. Other treatments involving anti-PD-1 options are also being further researched. Eggermont et al. investigated pembrolizumab treatment, finding a three-year progression-free survival rate of 32% (95% CI 25–40) when crossing over into treatment following the placebo [44]. With a plethora of emerging treatment course options, higher power trials are necessary to characterize the efficacy of each option. Benchmark studies between therapy option candidates are needed to identify the highest efficacy treatment method. 

**Figure 3:**
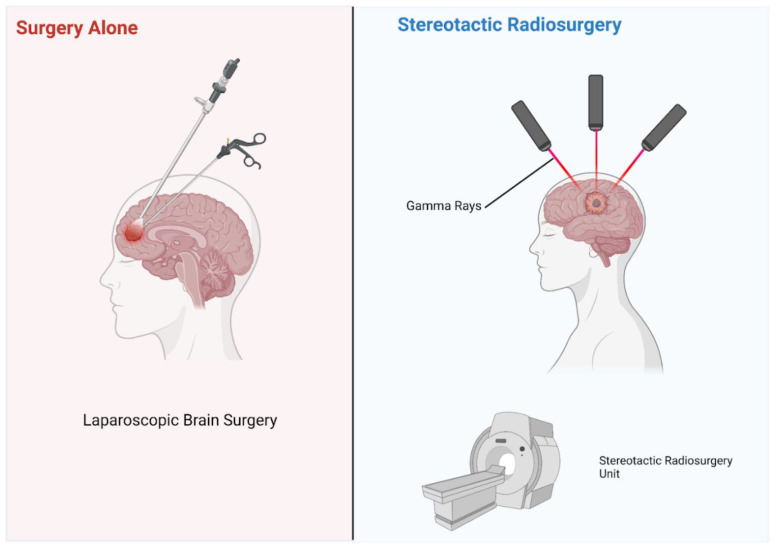
Illustration of two observational methods: radiosurgery and surgery alone. Figure created with BioRender.com (accessed on 18 December 2022).

In addition to improving drug therapy, emerging research addressing other clinical course features for patients with melanoma brain metastases is also abundant. Radiotherapy approaches, in particular, can often improve the accuracy and accessibility. A study conducted by Tran et al. assessed the pre-trial cost-effectiveness of whole-brain radiotherapy, hippocampal-avoidant radiotherapy, and observational methods such as stereotactic radiosurgery or surgery alone (Figure 3) [45]. Hippocampal avoidant radiotherapy was shown using probabilistic sensitivity analysis as the preferred method in 77% of simulations. Other studies have confirmed the data quality from observational methods such as whole brain radiotherapy and the preferred hippocampal avoidant radiotherapy [35,36]. Aside from the cost considerations, additional research addressing improvable features of utilized observational methods could lead to better patient outcomes and conserve clinical resources. Despite the magnitude of emerging research, further studies are needed to identify prospective front-line therapy options that promote greater clinical efficacy.

A review of 2762 studies of melanoma brain metastasis outlined several risk factors associated with hazard for death and survival benefit [46]. Hazard of death was associated with increased age, presence of extracranial metastasis, and an increased Charlson–Deyo score of 1 or 2 (comorbidity index) [46]. Increased survival was associated with government and private-based insurance and treatment at an academic center [46]. Furthermore, WBRT and WBRT + IT were associated with increased risk of death, whereas SRS + IT was associated with a survival benefit [46].

The BRAF gene plays a crucial role in determining patient treatment. Inhibitors of BRAF downstream MAPK and/or MEK are more effective than chemotherapy in the treatment of BRAFV600E-mutated metastatic melanoma [47]. However, the issue of drug resistance remains a significant challenge. A novel drug, E6201, acts as an ATP-competitive MEK1 inhibitor that has shown success in patients with brain metastasis with BRAF V600E and CTNNB1 mutations [47]. While the effect of CTNNB1 on the MEK pathway is unknown, E6201 provides insights into the mechanism of metastatic melanoma progression into brain metastasis [47]. A different study using patient derived xenografts highlighted RAS and MAP2K1/2 mutations as conferring resistance to BRAF inhibition [22]. Additionally, the established melanoma cell lines showed a significant bias toward BRAF, TP53 mutations, and CDKN2A loss [22]. Pre-clinical data by several groups have suggested that combining BRAF/MEK inhibitors with PI3K/mTOR inhibitors may overcome resistance in BRAF mutant melanomas [22]. A case study focused on BRAF G466E and BRAF K601E mutations with decreased and increased kinase activity, respectively. The results indicated that mutations resulting in increased MAP kinase activity may respond better to MEK inhibitors, whereas patients with mutations resulting in low MAP kinase activities may benefit from treatment with inhibitors of various immune checkpoints [48].

## 4. Discussion

The early detection and treatment are significant for MBM disease management [45,47]. MBM is typically detected in the later stages of cancer, which may be attributed to both the low permeability of the BBB and the lack of reliable biomarkers (at the time of writing) [17,48,49]. Currently, the biomarkers under study for early diagnosis include BRAFV600-Mutant ctDNA, LDH, and IGF-I [13]. The efficacy of LDH is under continued investigation with conflicting reports on the survival rates of patients with low versus high levels. There is less debate regarding the potential of the BRAF mutation. Higher rates of BRAF mutations were reported among MBM compared to primary and extracranial melanoma metastases [49]. Similar findings outside the data reviewed reported larger cases of MBMs and worse neurological symptoms among patients with tumor BRAF mutations compared to their wild-type counterparts [45]. The BRAF^V600^ mutation correlates with hyperactivation of the MAPK signaling pathway. Biomarkers are a viable option for the diagnosis of MBM and further studies implicate the NRAS and CKIT mutation as indicators, though unreliable markers [50,51]. Detection imaging through WB-DWI had a lower SE in detecting metastases and was reported to be effective at detecting extracranial metastasis [23]. MRI remains the preferred method for detecting brain lesions [22]. Although MRI is not considered effective among patients with completely resected stage III melanoma prior to administering adjuvant treatment [52], an awareness of the risk factors aids in early detection. Currently, MBM is reported to favor left-side tumors with a significantly higher risk in males than females [53].

Treatment combining targeted and immunotherapies with radiotherapy appears optimal for OS and PFS in patients with MBM, though co-administration is linked to adverse effects [40,54,55]. The combination of treatments is associated with increased radiation necrosis and grade 3/4 toxicities [55]. Incorporating radiotherapy, SRS is becoming preferred compared to WBRT due to its precise approach targeting metastatic tumors and minimizing adverse effects such as memory loss, as seen with WBRT [24,33]. The combination of BRAF + MEK inhibitors was shown to yield superior outcomes and prolong OS [49,50]. The addition of trametinib has been reported to improve OS compared to dabrafenib alone in patients with BRAF Val600 mutation-positive melanoma [56,57]. A second-line therapy of combined BRAF + MEK displayed the highest survival outcome when relative to immune checkpoint blockade therapy [50]. The most optimal treatment appears to be a combination including SRS and immunotherapy.

Clinical trials investigating emerging therapies are focused on immune checkpoint inhibitors and small-molecule targeted drugs. Temozolomide, in combination with sorafenib, has exhibited antiangiogenic effects indicating treatment potential [27]. Moreover, the co-administration of temozolomide and IFN-α2b showed improved responses and survival [26]. Additional immunotherapies including nivolumab and ipilimumab need further investigation regarding the optimal combination ratio and timing of treatment administration. The administration of ipilimumab alone improved OS, but may be enhanced with nivolumab [58]. There is a reported high intracranial response across cohorts of MBM patients treated with nivolumab and ipilimumab [40]. These reports are concurrent to those of patients responding with over 80% tumor regression following combined ipilimumab and nivolumab therapies [59]. Prolgolimab has also been identified as a potential therapy for non-resectable, metastatic melanoma patients [43]. Similarly, metastatic melanoma tumors have been reported to reduce in size following treatment with anti-PD-1 therapy through activated CD8+ T cells at the tumor site [60,61,62]. Anti-PD-1/L1 therapy minimized MBM and improved overall survival in mice with robust T cells in extracranial and intracranial metastatic sites when sequenced prior to MAPKi [61]. MEKi and anti-PD1 treatment in the BRAF wild-type subgroup induced a high tumor control rate of 83% and a median PFS of 7.1 months [63]. This combination of anti-PD1 with BRAFi and/or MAPKi requires further prospective studies. There is no reported difference in OS between first-line BRAF^V600^ and MEK inhibitors when compared to nivolumab + ipilimumab treatment, though both showed an improvement among patients [64]. This contradicts the reports of second-line ipilimumab + nivolumab following BRAF-MEKi, which showed poor activity, with MBM resisting treatment among both lines [64]. Emerging preclinical data for inhibitor buparlisib treatment was shown to be effective in mouse models [65]. Buparlisib alone displayed reduced proliferation, tumor progression, and induced apoptosis among the tissue samples. It is important to note that these findings were found in the brain tissues of treatment-naïve samples [65].

Though early detection is necessary to improve the efficacy of disease management, combined treatments appear to be most promising for improving the ongoing approaches [47,48]. Currently, therapies are incorporated into treatment courses regardless of surgical intervention; therefore, it is vital to ensure their efficacy. Further investigation into early detection and risk factors is necessary to improve the treatment and survival rates among patients with MBM, as brain metastasis is responsible for more than half of all melanoma deaths [65]. Objective treatment plans cannot be listed as each patient must receive specific treatment based on their medical history and preferences. 

## 5. Limitations

A primary limitation of the present review was the lack of available original literature meeting the established criteria set. Furthermore, the severity of melanoma that metastasizes to the brain limits the number of cases available to be studied and the time frame in which researchers have to test courses of treatment [15]. These limitations may pose issues in procuring cohorts that meet the study criteria and in gathering results from longitudinal studies exceeding six months [15]. Additionally, while ctDNA is a promising biomarker for predicting the course of metastatic melanoma, there exists evidence highlighting difficulties in its detectability in some patients weeks after the onset of treatment [17]. As a result of acquired resistance, solely looking at BRAFV600-mutant ctDNA may not represent a sufficient avenue to predict the clinical outcomes of advanced melanoma [17]. Existing studies are also yet to discern between the adverse effects of administered immunotherapies versus those associated with disease toxicities due to a lack of information on the timing of administration [55]. This information is needed to differentiate the sources of outcomes such as gangrene and impaired cognitive function [55]. The combination of immunotherapy treatments such as dabrafenib and trametinib showed promising results in increasing patient survival, as reported by Long and colleagues [18]. Overall, these and the other discussed drug combinations were tolerated well, but poor responsiveness observed in a subset of patients validates the demand for greater granularity of patient information in pooled research moving forward [17]. Future research should thus aim to isolate risk factors (genomic and otherwise) that may potentiate misjudged hazards associated with prospective immune therapy cocktails.

## 6. Conclusions

The present review identified current and emerging detection methods and treatments for MBM. Across sources, detection was reported to be predominantly through imaging including WB-DWI and MRI. Biomarkers were noted as potential indicators, though they are not currently reliable. The present treatment mainly relies on chemotherapies and targeted therapies as the BBB obstructs the success of other treatment methods. Approaches combining detection methods or treatments enhance the efficacy of both. The optimal ratio and timing of conjunctive therapies remain under investigation, though this administration method is associated with adverse effects. The patient survival rate and quality of life may improve with the minimization of the negative effects associated with combined treatment. Further investigation into the early detection of MBM—which may be attained with reliable biomarkers—would be most effective for disease management. With this, an objective course for care cannot be described as each patient requires personalized treatment based on their medical history and preference. The continued exploration of melanoma must include metastatic melanoma to improve the efficacy of detection and treatment. Overall, sources that include MBM point toward progress and patient benefits regarding risk, detection, survival, and treatment.

## Figures and Tables

**Table 1:** Summary of the risk factors that are independently associated with MBM progression-free survival.

Factors Associated with Lower PFS	Factors Associated with Increased PFS
ECOG ≳ 1	ECOG = 0
Elevated Serum LDH	LDH < ULN
≳3 Sites of Metastasis	<3 Sites of Metastasis
Non-Naïve Status	No Prior Treatment

## Data Availability

Not applicable.

## References

[1] Saginala K., Barsouk A., Aluru J.S., Rawla P., Barsouk A. (2021). Epidemiology of Melanoma. Med. Sci..

[2] Erdei E., Torres S.M. (2010). A new understanding in the epidemiology of melanoma. Expert Rev. Anticancer Ther..

[3] Bellenghi M., Puglisi R., Pontecorvi G., De Feo A., Carè A., Mattia G. (2020). Sex and Gender Disparities in Melanoma. Cancers.

[4] Eddy K., Shah R., Chen S. (2021). Decoding Melanoma Development and Progression: Identification of Therapeutic Vulnerabilities. Front. Oncol..

[5] Siegel R.L., Miller K.D., Fuchs H.E., Jemal A. (2021). Cancer Statistics, 2021. CA Cancer J. Clin..

[6] Damsky J.W.E., Rosenbaum L.E., Bosenberg M. (2010). Decoding Melanoma Metastasis. Cancers.

[7] Ajithkumar T., Parkinson C., Fife K., Corrie P., Jefferies S. (2015). Evolving treatment options for melanoma brain metastases. Lancet Oncol..

[8] Oliva I.G., Tawbi H., Davies M.A. (2017). Melanoma Brain Metastases: Current Areas of Investigation and Future Directions. Cancer J..

[9] Davies M.A., Liu P., McIntyre S., Kim K.B., Papadopoulos N., Hwu W.-J., Hwu P., Bedikian A. (2011). Prognostic factors for survival in melanoma patients with brain metastases. Cancer.

[10] Vosoughi E., Lee J.M., Miller J.R., Nosrati M., Minor D.R., Abendroth R., Lee J.W., Andrews B.T., Leng L.Z., Wu M. (2018). Survival and clinical outcomes of patients with melanoma brain metastasis in the era of checkpoint inhibitors and targeted therapies. BMC Cancer.

[11] Middleton M., Grob J.-J., Aaronson N., Fierlbeck G., Tilgen W., Seiter S., Gore M., Aamdal S., Cebon J., Coates A. (2000). Randomized phase III study of temozolomide versus dacarbazine in the treatment of patients with advanced metastatic malignant melanoma. J. Clin. Oncol..

[12] Rieth J., Swami U., Mott S., Zanaty M., Henry M., Bossler A., Greenlee J., Zakharia Y., Vanneste M., Jennings B. (2021). Melanoma Brain Metastases in the Era of Targeted Therapy and Checkpoint Inhibitor Therapy. Cancers.

[13] Page M.J., McKenzie J.E., Bossuyt P.M., Boutron I., Hoffmann T.C., Mulrow C.D., Shamseer L., Tetzlaff J.M., Akl E.A., Brennan S.E. (2021). The PRISMA 2020 statement: An updated guideline for reporting systematic reviews. BMJ (Clin. Res. Ed.).

[14] Hauswald H., Habl G., Krug D., Kehle D., Combs S., Bermejo J.L., Debus J., Sterzing F. (2013). Whole brain helical Tomotherapy with integrated boost for brain metastases in patients with malignant melanoma–a randomized trial. Radiat. Oncol..

[15] Samlowski W.E., Moon J., Witter M., Atkins M.B., Kirkwood J.M., Othus M., Ribas A., Sondak V.K., Flaherty L.E. (2017). High frequency of brain metastases after adjuvant therapy for high-risk melanoma. Cancer Med..

[16] Fuentes R., Osorio D., Hernandez J.E., Simancas-Racines D., Martinez-Zapata M.J., Cosp X.B. (2018). Surgery versus stereotactic radiotherapy for people with single or solitary brain metastasis. Cochrane Database Syst. Rev..

[17] Syeda M.M., Wiggins J.M., Corless B.C., Long G.V., Flaherty K.T., Schadendorf D., Nathan P.D., Robert C., Ribas A., Davies M.A. (2021). Circulating tumour DNA in patients with advanced melanoma treated with dabrafenib or dabrafenib plus trametinib: A clinical validation study. Lancet Oncol..

[18] Long G.V., Grob J.-J., Nathan P., Ribas A., Robert C., Schadendorf D., Lane S.R., Mak C., Legenne P., Flaherty K.T. (2016). Factors predictive of response, disease progression, and overall survival after dabrafenib and trametinib combination treatment: A pooled analysis of individual patient data from randomised trials. Lancet Oncol..

[19] Azam F., Latif M.F., Farooq A., Tirmazy S.H., AlShahrani S., Bashir S., Bukhari N. (2019). Performance Status Assessment by Using ECOG (Eastern Cooperative Oncology Group) Score for Cancer Patients by Oncology Healthcare Professionals. Case Rep. Oncol..

[20] Hess L.M., Brnabic A., Mason O., Lee P., Barker S. (2019). Relationship between Progression-free Survival and Overall Survival in Randomized Clinical Trials of Targeted and Biologic Agents in Oncology. J. Cancer.

[21] Dutriaux C., Robert C., Grob J.-J., Mortier L., Dereure O., Lebbe C., Mansard S., Grange F., Neidhardt E.-M., Lesimple T. (2022). An open label, non-randomised, phase IIIb study of trametinib in combination with dabrafenib in patients with unresectable (stage III) or distant metastatic (stage IV) BRAF V600-mutant melanoma: A subgroup analysis of patients with brain metastases. Eur. J. Cancer.

[22] Krepler C., Sproesser K., Brafford P., Beqiri M., Garman B., Xiao M., Shannan B., Watters A., Perego M., Zhang G. (2017). A Comprehensive Patient-Derived Xenograft Collection Representing the Heterogeneity of Melanoma. Cell Rep..

[23] Petralia G., Padhani A., Summers P., Alessi S., Raimondi S., Testori A., Bellomi M. (2013). Whole-body diffusion-weighted imaging: Is it all we need for detecting metastases in melanoma patients?. Eur. Radiol..

[24] Fogarty G., Morton R.L., Vardy J., Nowak A.K., Mandel C., Forder P.M., Hong A., Hruby G., Burmeister B., Shivalingam B. (2011). Whole brain radiotherapy after local treatment of brain metastases in melanoma patients—A randomised phase III trial. BMC Cancer.

[25] Pasquali S., Hadjinicolaou A.V., Sileni V.C., Rossi C.R., Mocellin S. (2018). Systemic treatments for metastatic cutaneous melanoma. Cochrane Database Syst. Rev..

[26] Richtig E., Hofmann-Wellenhof R., Pehamberger H., Forstinger C., Wolff K., Mischer P., Raml J., Fritsch P., Zelger B., Ratzinger G. (2004). Temozolomide and interferon alpha2b in metastatic melanoma stage IV. Br. J. Dermatol..

[27] Amaravadi R.K., Schuchter L.M., McDermott D.F., Kramer A., Giles L., Gramlich K., Carberry M., Troxel A.B., Letrero R., Nathanson K.L. (2009). Phase II Trial of Temozolomide and Sorafenib in Advanced Melanoma Patients with or without Brain Metastases. Clin. Cancer Res..

[28] Chiarion-Sileni V., Guida M., Ridolfi L., Romanini A., Del Bianco P., Pigozzo J., Brugnara S., Colucci G., Ridolfi R., De Salvo G.L. (2011). Central nervous system failure in melanoma patients: Results of a randomised, multicentre phase 3 study of temozolomide- and dacarbazine- based regimens. Br. J. Cancer.

[29] Gritsch D.M., Mrugala M.M.M., Marks L.A.M., Wingerchuk D.M.M., O’Carroll C.B.M. (2022). In Patients With Melanoma Brain Metastases, Is Combination Immune Checkpoint Inhibition a Safe and Effective First-Line Treatment? A Critically Appraised Topic. Neurologist.

[30] Guo T., Chu L., Chu X., Yang X., Li Y., Zhou Y., Xu D., Zhang J., Wang S., Hu J. (2022). Brain metastases, patterns of intracranial progression, and the clinical value of upfront cranial radiotherapy in patients with metastatic non-small cell lung cancer treated with PD-1/PD-L1 inhibitors. Transl. Lung Cancer Res..

[31] Bedikian A.Y., Richards J., Kharkevitch D., Atkins M.B., Whitman E., Gonzalez R. (2010). A phase 2 study of high-dose Allovectin-7 in patients with advanced metastatic melanoma. Melanoma Res..

[32] Li S., Wu X., Chen P., Pei Y., Zheng K., Wang W., Qiu E., Zhang X. (2019). Interferon-α versus interleukin-2 in Chinese patients with malignant melanoma: A randomized, controlled, trial. Anti-Cancer Drugs.

[33] Hong A.M., Fogarty G.B., Dolven-Jacobsen K., Burmeister B.H., Lo S.N., Haydu L.E., Vardy J.L., Nowak A., Dhillon H.M., Ahmed T. (2019). Adjuvant Whole-Brain Radiation Therapy Compared With Observation After Local Treatment of Melanoma Brain Metastases: A Multicenter, Randomized Phase III Trial. J. Clin. Oncol..

[34] Tsao M.N., Xu W., Wong R.K., Lloyd N., Laperriere N., Sahgal A., Rakovitch E., Chow E. (2018). Whole brain radiotherapy for the treatment of newly diagnosed multiple brain metastases. Cochrane Database Syst. Rev..

[35] Martinage G., Hong A.M., Fay M., Thachil T., Roos D., Williams N., Lo S., Fogarty G. (2018). Quality assurance analysis of hippocampal avoidance in a melanoma whole brain radiotherapy randomized trial shows good compliance. Radiat. Oncol..

[36] Fogarty G.B., Hong A., Dolven-Jacobsen K., Reisse C.H., Burmeister B., Haydu L.H., Dhillon H., Steel V., Shivalingam B., Drummond K. (2015). First interim analysis of a randomised trial of whole brain radiotherapy in melanoma brain metastases confirms high data quality. BMC Res. Notes.

[37] Janavicius M., Lachej N., Anglickiene G., Vincerzevskiene I., Brasiuniene B. (2020). Outcomes of Treatment for Melanoma Brain Metastases. J. Ski. Cancer.

[38] Kirkpatrick J.P., Wang Z., Sampson J.H., McSherry F., Herndon J.E., Allen K.J., Duffy E., Hoang J.K., Chang Z., Yoo D.S. (2015). Defining the optimal planning target volume in image-guided stereotactic radiosurgery of brain metastases: Results of a randomized trial. Int. J. Radiat. Oncol..

[39] Tétu P., Allayous C., Oriano B., Dalle S., Mortier L., Leccia M., Guillot B., Dalac S., Dutriaux C., Lacour J.-P. (2019). Impact of radiotherapy administered simultaneously with systemic treatment in patients with melanoma brain metastases within MelBase, a French multicentric prospective cohort. Eur. J. Cancer.

[40] Long G.V., Atkinson V., Lo S., Sandhu S., Guminski A.D., Brown M.P., Wilmott J.S., Edwards J., Gonzalez M., Scolyer R.A. (2018). Combination nivolumab and ipilimumab or nivolumab alone in melanoma brain metastases: A multicentre randomised phase 2 study. Lancet Oncol..

[41] Di Giacomo A.M., Chiarion-Sileni V., Del Vecchio M., Ferrucci P.F., Guida M., Quaglino P., Guidoboni M., Marchetti P., Cutaia O., Amato G. (2021). Primary Analysis and 4-Year Follow-Up of the Phase III NIBIT-M2 Trial in Melanoma Patients with Brain Metastases. J. Clin. Oncol..

[42] Larkin J., Weber J., Del Vecchio M., Gogas H., Arance A.M., Dalle S., Cowey C.L., Schenker M., Grob J.-J., Chiarion-Sileni V. (2022). Adjuvant nivolumab versus ipilimumab (CheckMate 238 trial): Reassessment of 4-year efficacy outcomes in patients with stage III melanoma per AJCC-8 staging criteria. Eur. J. Cancer.

[43] Tjulandin S., Demidov L., Moiseyenko V., Protsenko S., Semiglazova T., Odintsova S., Zukov R., Lazarev S., Makarova Y., Nechaeva M. (2021). Novel PD-1 inhibitor prolgolimab: Expanding non-resectable/metastatic melanoma therapy choice. Eur. J. Cancer.

[44] Eggermont A.M., Meshcheryakov A., Atkinson V., Blank C.U., Mandala M., Long G.V., Barrow C., Di Giacomo A.M., Fisher R., Sandhu S. (2021). Crossover and rechallenge with pembrolizumab in recurrent patients from the EORTC 1325-MG/Keynote-054 phase III trial, pembrolizumab versus placebo after complete resection of high-risk stage III melanoma. Eur. J. Cancer.

[45] Tran A.D., Fogarty G., Nowak A.K., Diaby V., Hong A., Watts C., Morton R.L. (2020). Cost-Effectiveness of Subsequent Whole-Brain Radiotherapy or Hippocampal-Avoidant Whole-Brain Radiotherapy Versus Stereotactic Radiosurgery or Surgery Alone for Treatment of Melanoma Brain Metastases. Appl. Health Econ. Health Policy.

[46] Caulfield J.I., Kluger H.M. (2022). Emerging Studies of Melanoma Brain Metastasis. Curr. Oncol. Rep..

[47] Larkin J.R., Dickens A.M., Claridge T.D.W., Bristow C., Andreou K., Anthony D.C., Sibson N.R. (2016). Early Diagnosis of Brain Metastases Using a Biofluids-Metabolomics Approach in Mice. Theranostics.

[48] Babiker H.M., Byron S.A., Hendricks W.P.D., Elmquist W.F., Gampa G., Vondrak J., Aldrich J., Cuyugan L., Adkins J., De Luca V. (2019). E6201, an intravenous MEK1 inhibitor, achieves an exceptional response in BRAF V600E-mutated metastatic malignant melanoma with brain metastases. Investig. New Drugs.

[49] Richtig G., Aigelsreiter A., Kashofer K., Talakic E., Kupsa R., Schaider H., Richtig E. (2016). Two case reports of rare BRAF mutations in exon 11 and exon 15 with discussion of potential treatment options. Case Rep. Oncol..

[50] Franklin C., Mohr P., Bluhm L., Grimmelmann I., Gutzmer R., Meier F., Garzarolli M., Weichenthal M., Pfoehler C., Herbst R. (2022). Impact of radiotherapy and sequencing of systemic therapy on survival outcomes in melanoma patients with previously untreated brain metastasis: A multicenter DeCOG study on 450 patients from the prospective skin cancer registry ADOREG. J. Immunother. Cancer.

[51] In G.K., Poorman K., Saul M., O’Day S., Farma J.M., Olszanski A.J., Gordon M.S., Thomas J.S., Eisenberg B., Flaherty L. (2020). Molecular profiling of melanoma brain metastases compared to primary cutaneous melanoma and to extracranial metastases. Oncotarget.

[52] Derks S., de Joode K., Mulder E., Ho L., Joosse A., de Jonge M., Verhoef C., Grünhagen D., Smits M., Bent M.V.D. (2022). The meaning of screening: Detection of brain metastasis in the adjuvant setting for stage III melanoma. ESMO Open.

[53] Tan X.-L., Le A., Tang H., Brown M., Scherrer E., Han J., Jiang R., Diede S.J., Shui I.M. (2022). Burden and Risk Factors of Brain Metastases in Melanoma: A Systematic Literature Review. Cancers.

[54] Jablonska P.A., Fong C.H., Kruser T., Weiss J., Liu Z.A., Takami H., Narita Y., de Moraes F.Y., Dasgupta A., Ong C.K. (2022). Recommended first-line management of brain metastases from melanoma: A multicenter survey of clinical practice. Radiother. Oncol. J. Eur. Soc. Ther. Radiol. Oncol..

[55] van Opijnen M.P., Dirven L., Coremans I.E., Taphoorn M.J., Kapiteijn E.H. (2019). The impact of current treatment modalities on the outcomes of patients with melanoma brain metastases: A systematic review. Int. J. Cancer.

[56] Tawbi H.A., Boutros C., Kok D., Robert C., McArthur G. (2018). New Era in the Management of Melanoma Brain Metastases. Am. Soc. Clin. Oncol. Educ. Book.

[57] Long G.V., Stroyakovskiy D., Gogas H., Levchenko E., de Braud F., Larkin J., Garbe C., Jouary T., Hauschild A., Grob J.-J. (2015). Dabrafenib and trametinib versus dabrafenib and placebo for Val600 BRAF-mutant melanoma: A multicentre, double-blind, phase 3 randomised controlled trial. Lancet.

[58] Hodi F.S., O’Day S.J., McDermott D.F., Weber R.W., Sosman J.A., Haanen J.B., Gonzalez R., Robert C., Schadendorf D., Hassel J.C. (2010). Improved survival with ipilimumab in patients with metastatic melanoma. N. Engl. J. Med..

[59] Wolchok J.D., Kluger H., Callahan M.K., Postow M.A., Rizvi N.A., Lesokhin A.M., Segal N.H., Ariyan C.E., Gordon R.-A., Reed K. (2013). Nivolumab plus ipilimumab in advanced melanoma. N. Engl. J. Med..

[60] Tumeh P.C., Harview C.L., Yearley J.H., Shintaku I.P., Taylor E.J.M., Robert L., Chmielowski B., Spasic M., Henry G., Ciobanu V. (2014). PD-1 blockade induces responses by inhibiting adaptive immune resistance. Nature.

[61] Wang Y., Liu S., Yang Z., Algazi A.P., Lomeli S.H., Wang Y., Othus M., Hong A., Wang X., Randolph C.E. (2021). Anti-PD-1/L1 lead-in before MAPK inhibitor combination maximizes antitumor immunity and efficacy. Cancer Cell.

[62] Huynh S., Mortier L., Dutriaux C., Maubec E., Boileau M., Dereure O., Leccia M.-T., Arnault J.P., Brunet-Possenti F., Aubin F. (2020). Combined Therapy with Anti-PD1 and BRAF and/or MEK Inhibitor for Advanced Melanoma: A Multicenter Cohort Study. Cancers.

[63] Lau P.K.H., Feran B., Smith L., Lasocki A., Molania R., Smith K., Weppler A., Angel C., Kee D., Bhave P. (2021). Melanoma brain metastases that progress on BRAF-MEK inhibitors demonstrate resistance to ipilimumab-nivolumab that is associated with the Innate PD-1 Resistance Signature (IPRES). J. Immunother. Cancer.

[64] Amaral T., Kiecker F., Schaefer S., Stege H., Kaehler K., Terheyden P., Gesierich A., Gutzmer R., Haferkamp S., Uttikal J. (2020). Combined immunotherapy with nivolumab and ipilimumab with and without local therapy in patients with melanoma brain metastasis: A DeCOG* study in 380 patients. J. Immunother. Cancer.

[65] Amaral T., Niessner H., Sinnberg T., Thomas I., Meiwes A., Garbe C., Garzarolli M., Rauschenberg R., Eigentler T., Meier F. (2020). An open-label, single-arm, phase II trial of buparlisib in patients with melanoma brain metastases not eligible for surgery or radiosurgery—The BUMPER study. Neuro-Oncol. Adv..

